# The Risk for Glucose Intolerance after Gestational Diabetes Mellitus since the Introduction of the IADPSG Criteria: A Systematic Review and Meta-Analysis

**DOI:** 10.3390/jcm8091431

**Published:** 2019-09-10

**Authors:** Katrien Benhalima, Karen Lens, Jan Bosteels, Mathieu Chantal

**Affiliations:** 1 Department of Endocrinology, University Hospital Gasthuisberg, KU Leuven, Herestraat 49, 3000 Leuven, Belgium; 2 Medical School, University Hospital Gasthuisberg, KU Leuven, Herestraat 49, 3000 Leuven, Belgium; 3 Department of Obstetrics & Gynecology, Imelda Ziekenhuis, Imeldalaan 9, 2820 Bonheiden, Belgium

**Keywords:** gestational diabetes mellitus, International Association of Diabetes and Pregnancy Study Groups (IADPSG), postpartum, type 2 diabetes, impaired glucose tolerance, glucose intolerance, stroke, myocardial infarction, diagnostic criteria

## Abstract

The aim of the study was to assess the postpartum risk for glucose intolerance since the introduction of the ‘International Association of Diabetes and Pregnancy Study Groups’ (IADPSG) criteria for gestational diabetes mellitus (GDM). Studies published since 2010 were included, which evaluated the risk for type 2 diabetes mellitus (T2DM), impaired glucose tolerance (IGT), and cardiovascular (CV) events in women with previous GDM compared to normal glucose tolerant women. We included forty-three studies, evaluating 4,923,571 pregnant women of which 5.8% (284,312) had a history of GDM. Five studies used IADPSG criteria (*n* = 6174 women, 1314 with GDM). The overall pooled relative risk (RR) for postpartum T2DM was 7.42 (95% CI: 5.99–9.19) and the RR for postpartum T2DM with IADPSG criteria was 6.45 (95% CI: 4.74–8.77) compared to the RR of 9.08 (95% CI: 6.96–11.85; *p* = 0.17) for postpartum T2DM based on other diagnostic criteria. The RR for postpartum IGT was 2.45 (95% CI: 1.92–3.13), independent of the criteria used. None of the available studies with IADPSG criteria evaluated the risk for CV events. Women with a history of GDM based on the IADPSG criteria have a similarly increased risk for postpartum glucose intolerance compared to GDM based on other diagnostic criteria. More studies with GDM based on the IADPSG criteria are needed to increase the quality of evidence concerning the long-term metabolic risk.

## 1. Introduction

Gestational diabetes mellitus (GDM) is defined as diabetes diagnosed in the second or third trimester of pregnancy provided that overt diabetes has been excluded in early pregnancy [1]. Women with a history of GDM are at an increased risk of developing type 2 diabetes mellitus (T2DM) postpartum and also have a significantly higher risk of developing a metabolic syndrome and cardiovascular diseases compared to women with normal glucose tolerance (NGT) in pregnancy [2,3]. The diagnostic criteria for GDM were initially based on the postpartum risk of developing T2DM, as proposed by the Carpenter and Coustan (CC) criteria, while the World Health Organization (WHO) initially defined GDM as impaired glucose tolerance (IGT) outside pregnancy [4]. Based on the “Hyperglycemia and Adverse Pregnancy Outcome” (HAPO) study, a universal one-step diagnostic approach with a 75 g oral glucose tolerance test (OGTT) and more stringent diagnostic criteria for GDM were proposed in 2010 by the “International Association of Diabetes and Pregnancy Study Groups” (IADPSG) [5,6]. The IADPSG criteria are the first diagnostic criteria for GDM based on adverse pregnancy outcomes and have now been endorsed by several national and international societies, including the WHO since 2013 [7]. A systematic review of 2009, published before the introduction of the IADPSG criteria, showed that women with GDM have a seven-fold increased risk of developing T2DM later in life compared to NGT women during pregnancy [2]. A subgroup analysis showed no difference in the postpartum risk for T2DM when women were stratified according to the different diagnostic criteria used for GDM [2]. However, this systematic review included mostly old studies and was performed before the introduction of the IADPSG criteria for GDM. A systematic review from 2018 included more recent studies and confirmed the seven-fold increased postpartum risk for T2DM but did not evaluate the postpartum risk based on the IADPSG criteria compared to other criteria [8]. The IADPSG screening strategy identifies women with milder degrees of hyperglycemia during pregnancy compared to other diagnostic criteria and screening strategies (two-step or selective screening), which might lead to a lower proportion at risk of postpartum glucose intolerance [9,10,11]. Our objective was therefore to assess the postpartum risk for glucose intolerance and cardiovascular events in women with GDM based on the IADPSG criteria compared to other diagnostic criteria.

## 2. Methods

### 2.1. Protocol and Registration

Data were reported in accordance with the Guidelines for Meta-Analyses and Systematic Reviews of Observational Studies (MOOSE) [12]. The study protocol was registered on the International Prospective Register of Systematic Reviews (PROSPERO) under the following identification number: CRD42018102315.

### 2.2. Data Sources and Searches

We searched MEDLINE, EMBASE, Web of Science, the Cochrane Library, and Latin American and Caribbean Health Sciences Literature (LILACS) up to 20 January 2019 for eligible studies published after 2009. Electronic searches were supplemented with manual searches of references of included studies. The journals Diabetes Care and Diabetologia (including special supplements) were screened for possible relevant articles. Table S1 shows our detailed search strategy. This was developed in consultation with biomedical reference librarians of the Catholic University of Leuven (KU Leuven).

### 2.3. Study Selection

We included retrospective and prospective cohort, case-control and cross-sectional studies that evaluated the postpartum risk of glucose intolerance (T2DM and IGT (defined as impaired fasting glycemia and/or impaired glucose tolerance based on the 2 h glycemia on the OGTT)) or cardiovascular events (myocardial infarction or stroke) in women with a history of GDM. The study selection was performed by two investigators (K.B. and K.L.) independently. To be included, studies had to be published after 2009 (after the introduction of the IADPSG criteria), have a minimum follow-up period of six weeks after the index pregnancy, and contain a control group without a history of GDM. The control NGT group could be matched or not matched to the GDM group. No matched controls were defined as NGT women not matched by other characteristics (such as age and body mass index (BMI)) to the GDM group. Studies were included independent of which GDM screening strategy or diagnostic criteria were used. Studies including women with type 1 diabetes, T2DM, or with overt diabetes in early pregnancy were excluded. No language restriction was applied, but studies written in languages other than English, French, or Spanish were only included if they could be adequately translated using Google Translate.

### 2.4. Data Extraction

Studies were uploaded in Endnote (version X9). Duplicated records were removed using Endnote software and by performing a manual search. Two investigators (K.B. and K.L.) independently reviewed and extracted relevant data from each included report using pre-designed forms. Any disagreement in data extraction was reconciled by consensus. If needed, a third reviewer (J.B.) was consulted. Extracted data included study setting, design, participant characteristics (country, origin, definition of control group, family history of T2DM, personal history of GDM, maternal age and body mass index (BMI, kg/m^2^) in the index pregnancy and at follow-up, and parity), characteristics about the diagnosis of GDM, IGT, and T2DM (timing of screening, screening strategy, diagnostic criteria), outcome data, and follow-up data (duration and loss to follow-up). The origin of women was considered to be mixed if <80% of the study participants had the same ethnicity. When studies reported data concerning the risk for T2DM at multiple time points after the index pregnancy, the data with the longest follow-up were extracted since the risk for glucose intolerance increases with the duration of the follow-up. Authors were contacted for missing outcome data.

### 2.5. Outcomes

Outcomes included T2DM as the primary outcome and IGT, myocardial infarction, and stroke as the secondary outcomes. No restrictions were applied concerning the strategy (fasting glycemia, OGTT, or HbA1c) or diagnostic criteria used to define glucose intolerance postpartum.

### 2.6. Data Synthesis and Analysis

Pooled analyses and subgroup analyses were conducted on Review Manager (RevMan), version 5.3 (The Nordic Cochrane Centre, The Cochrane Collaboration, Copenhagen, Denmark) using the DerSimonian and Laird random-effects model and Mantel–Haenszel methods, given their better statistical properties in case of sparse data, to calculate the summary relative risk (RR). Unadjusted absolute risks were determined. Heterogeneity was assessed using the Cochran Q statistic and quantified using the I^2^ statistic, where I^2^ > 50% was considered as important heterogeneity [13]. We investigated potential sources of heterogeneity by performing the following subgroup analyses for T2DM and IGT: duration of postpartum follow-up (<3 years, ≥3 to <6 years, ≥6 to <10 years, ≥10 to <15 years, ≥15 to <20 years, ≥20 years, or not reported), maternal age during pregnancy and at follow-up (<30 years, ≥30 to <35 years, ≥35 years, or not reported), maternal BMI during pregnancy and at follow-up (<25 kg/m^2^, ≥25 to <30 kg/m^2^, ≥30 kg/m^2^, or not reported), origin (White, Asian, Pacific Islanders, Middle Eastern, mixed, or not reported), diagnostic criteria, and screening strategy used for GDM and glucose intolerance postpartum. Heterogeneity within and between subgroups was assessed using the I^2^-test, with a significance level of 0.10.

Sensitivity analyses were performed to evaluate whether the conclusions would have differed if eligibility had been restricted to studies with good quality according to the Newcastle–Ottawa Scale (NOS) or prospective cohort studies.

### 2.7. Quality Assessment

The same two reviewers (K.B. and K.L.) independently evaluated the quality of the studies in accordance with NOS [14]. The quality assessment was divided into good quality (three or four stars for selection, one or two stars for comparability, and two or three stars for outcome/exposure), fair quality (two stars for selection, one or two stars for comparability, and two or three stars for outcome/exposure), and poor quality (none or one star for selection, no stars for comparability, or none or one star for outcome/exposure). In order to investigate possible publication bias, we performed a visual inspection of funnel plots.

## 3. Results

### 3.1. Search Results

**Figure 1:**
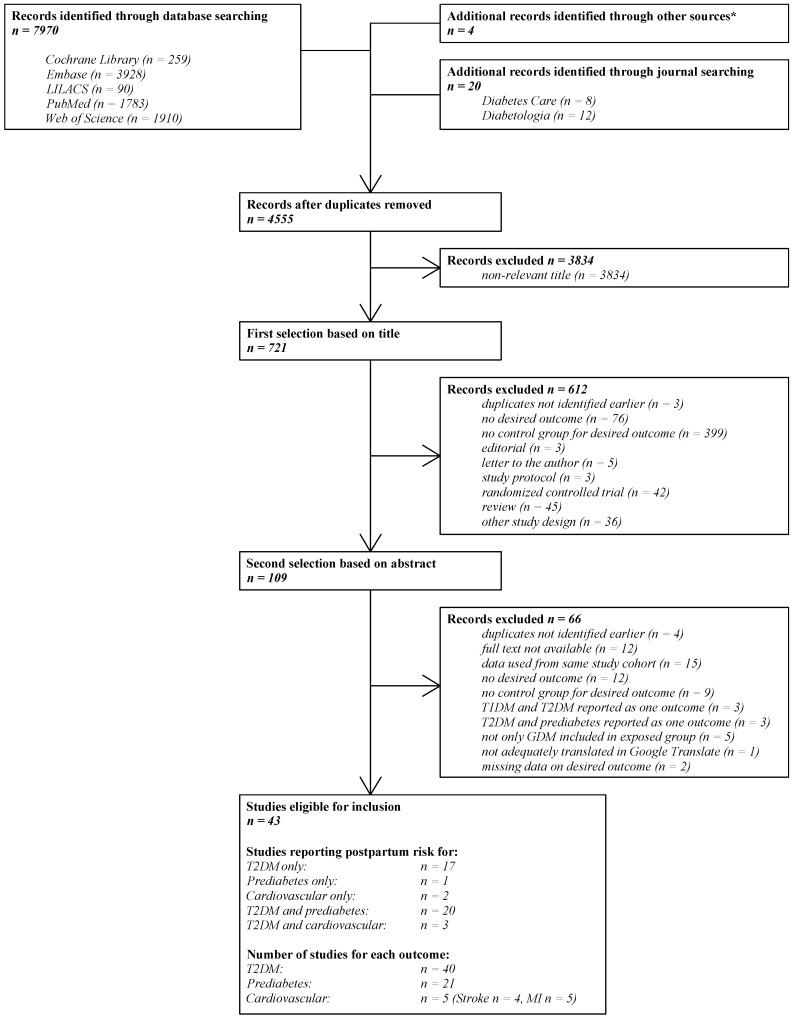
The literature search and selection process. N: the number of studies; LILACS: Latin American and Caribbean Health Sciences Literature; T1DM: type 1 diabetes mellitus; T2DM: type 2 diabetes mellitus; GDM: gestational diabetes mellitus; MI: myocardial infarction. * Retrieved from the systematic review of Song et al. [8].

Figure 1 shows the literature search and selection process. Our initial search yielded 7970 publications, of which 109 articles were reviewed in full and 43 studies were included in the final analysis [3,11,15,16,17,18,19,20,21,22,23,24,25,26,27,28,29,30,31,32,33,34,35,36,37,38,39,40,41,42,43,44,45,46,47,48,49,50,51,52,53,54,55]. Forty studies reported on T2DM [3,11,15,16,17,18,19,20,21,22,23,24,25,26,27,28,29,30,31,32,33,34,35,36,37,38,39,40,41,42,43,44,45,46,47,48,49,50,51,55], twenty-one on IGT [11,15,19,20,21,22,23,24,25,26,27,28,29,30,31,32,33,34,39,53,55], and five studies on cardiovascular events [16,17,18,52,54].

### 3.2. Study Characteristics

Table 1 shows the characteristics of included studies. Across all studies, the total number of pregnant women included was 4,923,571, of which 284,312 had GDM (5.8%). Of all studies, 18 (41.9%) included less than 200 women with GDM. The follow-up ranged from 12 weeks to 25.7 years postpartum, with a mean follow-up of 7.9 years. Of all included studies, 19 (44.2%) were prospective cohorts, 13 (30.2%) retrospective cohorts, 6 (14.0%) case-control studies, and 5 (11.6%) cross-sectional studies. Fifteen studies (34.9%) were performed in Europe, ten (23.3%) in Asia, fourteen (32.6%) in North America, three (7.0%) in Australia, and one (2.3%) was an international study. The mean maternal age at follow-up was 36.5 years with a median BMI at follow-up of 26.9 kg/m^2^. The drop-out rate was only reported in six (14.0%) studies—three had a non important (<5%) drop-out and three studies reported a drop-out of >20%.

The diagnostic criteria for GDM were reported in 29 (67.4%) studies, of which six (14.0%) used the 1999 WHO criteria, five (11.6%) the IADPSG criteria, six (14.0%) the CC criteria, four (9.3%) the Canadian Diabetes Association (CDA) criteria, two (4.7%) the Australasian Diabetes in Pregnancy Society (ADIPS) criteria, two (4.7%) the National Diabetes Data Group (NDDG) criteria, one (2.3%) the German Diabetes Association criteria, and three (7.0%) used other local criteria. The five studies using the IADPSG screening strategy, included 6174 women of which 1314 had GDM, with a mean follow-up of 5.6 years, mean maternal age at follow-up of 36.9 years, and median maternal BMI at follow-up of 29.3 kg/m^2^. Three studies were performed in Europe, one in Asia, and one was an international study.

The screening strategy for GDM was reported in 24 (55.8%) studies, of which three (7.0%) used screening for GDM based on risk factors, 10 (23.3%) studies used a two-step screening strategy with a glucose challenge test (GCT), and 11 (25.6%) studies used a universal one-step approach with a 75 g OGTT. T2DM was defined according to the ADA criteria in 13 (32.5%) studies, the WHO criteria in seven (17.5%) studies, the CDA criteria in three (7.5%) studies, and the German Diabetes Association criteria in one study (2.5%). IGT was defined according to the ADA criteria in nine (42.9%) studies, the WHO criteria in five (23.8%) studies, and the CDA criteria in three (14.3%) studies.

### 3.3. Postpartum Risk of Type 2 Diabetes

**Figure 2:**
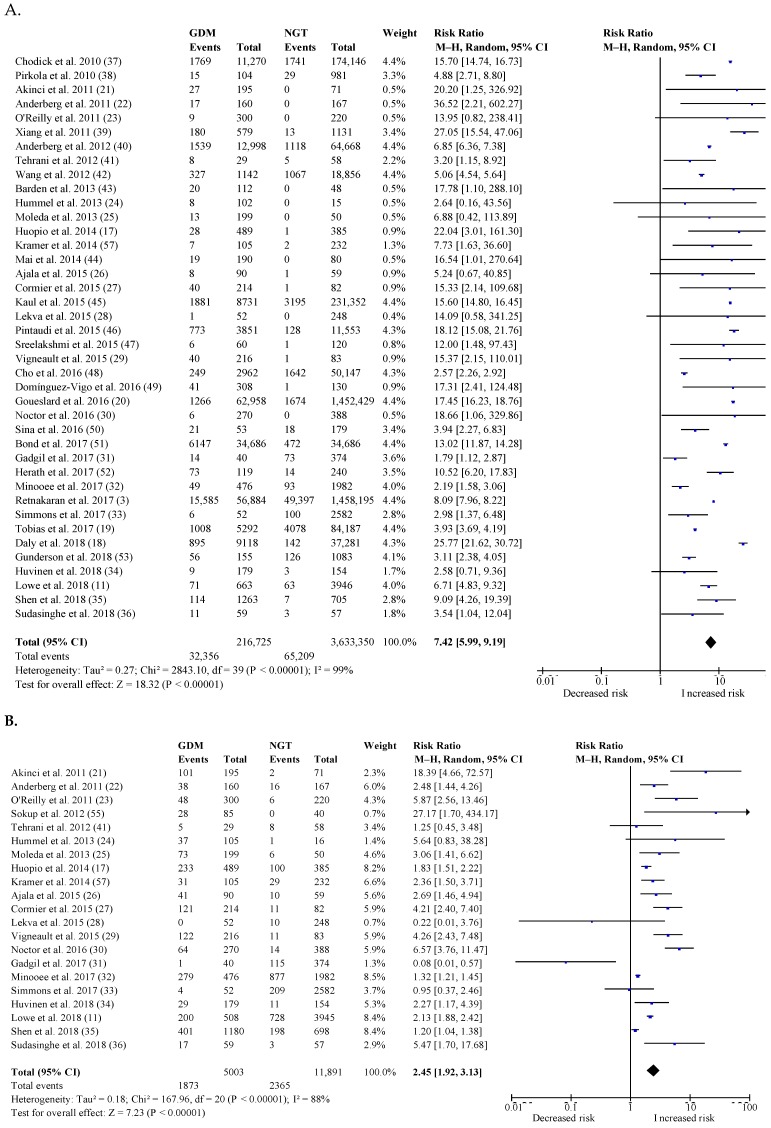

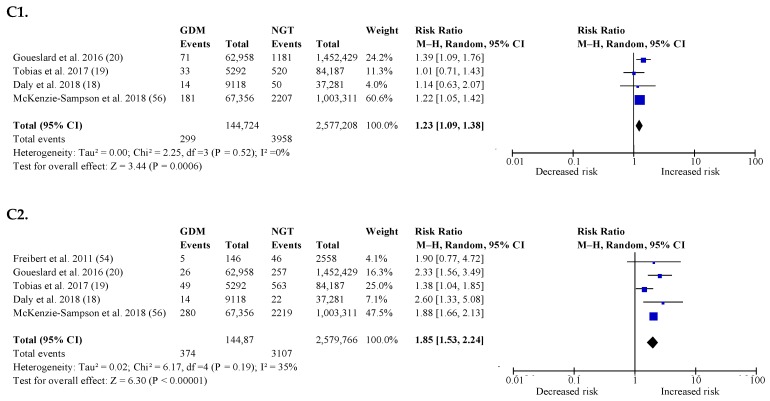
The overall postpartum risk for glucose intolerance and cardiovascular events in women with a history of GDM compared to NGT women: (**A**) Type 2 diabetes mellitus (T2DM), (**B**) prediabetes, (**C1**) stroke, and (**C2**) myocardial infarction. T2DM: Type 2 diabetes mellitus; GDM, gestational diabetes mellitus; 95% CI: confidence interval; M–H: Mantel–Haenszel method; df: degrees of freedom; RR: relative risk or risk ratio. X-axis is log scale of RR. Squares represent the RR of an individual study. The size of the square is proportional to the weight of the study, which was calculated using a random-effects model. The horizontal lines indicate 95% CI. The diamond represents the pooled RR with its 95% CI.

Figure 2A shows the overall postpartum risk for T2DM in women with a history of GDM compared to NGT women. The risk for T2DM stratified according to the IADPSG criteria compared to other diagnostic criteria is reported in Table 2A. The overall pooled RR for postpartum T2DM was 7.42 (95% CI: 5.99–9.19). The RR for postpartum T2DM for GDM based on the IADPSG criteria was 6.45 (95% CI: 4.74–8.77) compared to a RR of 9.08 (95% CI: 6.96–11.85; *p* = 0.17) for GDM based on other diagnostic criteria. There was substantial heterogeneity across different subgroups (Table 2A). The highest RR for T2DM was seen within the first 6 years after the index pregnancy. The risk for T2DM decreased with a longer follow-up period but remained significantly increased in comparison to NGT women. The RR was highest in women aged ≥30 to <35 years at follow-up, among women with a BMI ≥ 30 kg/m^2^ during pregnancy, in women with a BMI < 25 kg/m^2^ at follow-up, and in women with a White or mixed origin. The RR for postpartum T2DM was not significantly different according to the screening strategy used for GDM or the screening strategy used for T2DM postpartum.

### 3.4. Postpartum Risk of Impaired Glucose Tolerance

Figure 2B shows the overall postpartum risk for IGT in women with a history of GDM compared to NGT women. The risk for IGT stratified according to studies using the IADPSG criteria compared to other criteria is reported in Table 2B. The overall pooled RR for postpartum IGT was 2.45 (95% CI: 1.92–3.13). The RR for postpartum IGT for GDM based on the IADPSG criteria was 2.79 (95% CI: 1.30–5.96) compared to a RR of 2.03 (95% CI: 1.57–2.62; *p* = 0.73) for GDM based on other diagnostic criteria. Heterogeneity was substantial across different subgroups (Table 2B). The highest RR for IGT was seen within the first 3 years after the index pregnancy. The risk for IGT decreased with a longer follow-up period but remained significantly increased in comparison to NGT women. The RR was highest in women with a BMI ≥30 kg/m^2^ during pregnancy, in women with a BMI of 25–30 kg/m^2^ at follow-up, and in women with a White or mixed origin. The RR for IGT was not significantly different according to the screening strategy used for GDM or according to the diagnostic criteria used for IGT or maternal age during pregnancy or postpartum.

### 3.5. Sensitivity Analyses

Studies with good quality according to the NOS showed a much higher RR for T2DM of 15.21 (95% CI 8.36–27.69), while prospective cohort studies showed a lower RR for T2DM of 4.41 (95% CI: 3.64–5.35) compared to the overall effect (Table S3). Studies with good quality according to the NOS showed a higher RR for IGT of 4.57 (95% CI: 2.13–9.80), while prospective cohort studies showed a similar RR for IGT of 2.41 (95% CI: 1.87–3.10) as the overall effect (Table S2).

### 3.6. Postpartum Risk of Cardiovascular Events

Figure 2C shows the overall postpartum risk for stroke and myocardial infarction in women with a history of GDM compared to NGT women. RR for stroke was 1.23 (95% CI: 1.09–1.38) and RR for myocardial infarction was 1.85 (95% CI: 1.53–2.24). Of all studies evaluating cardiovascular events, none used the IADPSG criteria. Three studies reported on the postpartum risk of both T2DM and cardiovascular events [16,17,18]. However, only one study reported on the cardiovascular risk in women with previous GDM with and without T2DM and showed that women with GDM without T2DM had no increased risk (RR for stroke of 0.94 (95% CI: 0.63–1.41) and RR for myocardial infarction of 1.26 (95% CI: 0.90–1.76)), while women with GDM and T2DM had a significantly increased risk for myocardial infarction (RR of 1.28 (95% CI: 0.64–2.58) for stroke and RR for myocardial infarction of 1.93 (95% CI: 1.12–3.33)) [17].

### 3.7. Publication Bias

The funnel plots are shown in Figure S1. Since the estimated RRs for individual studies had a similar value, a visual inspection of the funnel plots for T2DM and cardiovascular events was not applicable. A visual inspection of the funnel plot for IGT suggested the absence of publication bias or only a small effect.

### 3.8. Assessment of Quality

Table S3 shows the quality of studies according to the NOS. Of all the 40 studies for T2DM, 30 (75.0%) had poor quality. The poor quality was mostly due to the absence of a matched control group. Of all the studies for IGT and cardiovascular events, 15 (71.4%) and 3 (60.0%), respectively, had poor quality.

## 4. Discussion

### 4.1. Summary of Findings

The present systematic review and meta-analysis of 43 studies, including nearly five million pregnant women of which 6% (about 280,000) had prior GDM, showed that women with a history of GDM based on the IADPSG criteria have a similarly increased risk for postpartum glucose intolerance compared to GDM based on other diagnostic criteria and other GDM screening strategies. Overall, women with GDM have a seven-fold increased risk of developing T2DM, a two-fold increased risk of developing IGT, and about a 1.5 times increased risk for cardiovascular events later in life compared to NGT women during pregnancy. This might be an underestimation, as the sensitivity analyses showed that the highest quality studies demonstrated a fifteen-fold increased risk for T2DM and a four-fold increased risk for IGT in women with a history of GDM compared to NGT women. The highest risk for developing glucose intolerance was present within the 3–6 years after the index pregnancy. In addition, the highest risk for T2DM was present in White and mixed-race women, in women with higher age and higher BMI during pregnancy, and in normal weight women at follow-up. The highest risk for IGT was present in obese women during pregnancy and in White and mixed-race women.

### 4.2. Results in Relation to What We Already Know

Our findings extend those of previous systematic reviews and meta-analyses showing that women with GDM have a seven-fold increased risk of developing T2DM later in life compared to NGT women during pregnancy [2,8]. The first systematic review of 2009 included twenty cohort studies and showed no significant heterogeneity from the subgroup analysis of the criteria used for the diagnosis of GDM and T2DM [2]. However, this systematic review included older studies and was performed before the introduction of the IADPSG criteria for GDM. In contrast to our systematic review, effect estimates were broadly consistent in all subgroups analyzed (such as ethnic origin, follow-up time, maternal age, and BMI) [2]. The most recent systematic review and meta-analysis from 2018 evaluated more than 2 million pregnant women from thirty cohort studies and confirmed the unadjusted seven-fold increased postpartum risk for T2DM. However, this review did not evaluate the postpartum risk based on the IADPSG criteria compared to other diagnostic criteria for GDM [8]. Twenty-eight cohorts reported the diagnostic criteria used for GDM but none of the included studies used the IADPSG criteria for GDM. In line with the results of our systematic review, the 2018 review showed that the highest risk for T2DM was present within 3–6 years after the index pregnancy and that women with normal weight at follow-up had the highest RR for T2DM [8]. As in our systematic review, women with a White origin had a higher RR of postpartum T2DM than Asian women. As in previous systematic reviews, no significant heterogeneity was observed according to the screening strategy used for GDM or according to the screening strategy and diagnostic criteria used for glucose intolerance postpartum [2,8].

To date, long-term data on the risk of T2DM developing in women with GDM diagnosed by the universal IADPSG screening strategy have been limited. In general, the IADPSG screening strategy identifies women with milder degrees of hyperglycemia during pregnancy compared to other diagnostic criteria and screening strategies (two-step or selective screening), which might lead to a lower proportion at risk of postpartum glucose intolerance [9,10,11]. We have previously shown glucose intolerance in 42% of women three months after a pregnancy with GDM diagnosed by a two-step screening strategy with a GCT, while less than 20% of women had glucose intolerance in early postpartum after a GDM pregnancy based on a universal one-step diagnostic approach with the IADPSG criteria [9,10]. A follow-up of the HAPO study 10–14 years postpartum showed that untreated women with GDM, defined post hoc by the IADPSG criteria, had significantly higher rates of a glucose metabolism disorder than women without GDM (52.2% vs. 20.1% (T2DM 10.7% vs. 1.6%)) [11]. However, GDM, according to the CC criteria, was associated with a much higher risk for T2DM (20% vs. 7.9%) compared to women with GDM according to the IADPSG criteria alone [11]. Our systematic review includes the largest number of studies to date on the risk for T2DM after GDM and presents the first summarized data on the risk for glucose intolerance postpartum in GDM based on the IADPSG criteria compared to GDM based on other diagnostic criteria. In addition, we have shown that women with previous GDM are also at increased risk of stroke and myocardial infarction. This is in line with a recent meta-analysis showing that women with GDM had a two-fold higher risk for future cardiovascular events and that this cardiovascular risk is independent of the intercurrent development of T2DM [56].

### 4.3. Implications

Our study has important public health implications. The IADPSG criteria are the first diagnostic criteria based on pregnancy outcomes. By implementing the IADPSG screening strategy, important adverse pregnancy outcomes can be prevented. However, due to concerns concerning the increased workload and associated costs, the adoption of the IADPSG screening strategy varies worldwide [57]. The limited data on the long-term risk for glucose intolerance postpartum in women with GDM based on the IADPSG criteria contribute to the ongoing discussion on whether implementing the IADPSG screening strategy will be cost-effective. Our data show that women with GDM based on the IADPSG criteria have a similarly increased risk for glucose intolerance postpartum compared to women with GDM based on other diagnostic criteria or other screening strategies for GDM. Since the implementation of the IADPSG screening strategy has led to an important increase in the prevalence of GDM, this might offer a window of opportunity to identify a large group of women at increased risk of glucose intolerance later in life. This can help to timely implement strategies to prevent the development of T2DM. Our data confirm that the highest RR to develop glucose intolerance is present within the first three to six years after the index pregnancy, highlighting the importance to start early after delivery with screening and prevention programs in this high-risk group to prevent and timely detect T2DM. In addition, continued follow-up beyond six years postpartum remains important since the risk to develop glucose intolerance over time remains significantly higher compared to women without a history of GDM. Our data also show that women with a White origin and women with normal weight at follow-up have the highest risks to develop T2DM. This might suggest that GDM identifies a high-risk group for T2DM at a young age due to beta-cell dysfunction and that genetic predisposition might play an important role in Caucasian women. However, caution for interpretation is warranted since many subgroup analyses were based on a limited number of studies and the majority of studies evaluated Caucasian women.

### 4.4. Strengths and Limitations

This systematic review and meta-analysis included the largest number of studies to date on the long-term risk for glucose intolerance after GDM. In contrast to other systematic reviews, our review included only recent studies published after 2009, and we did not only include cohort studies but also case-control and cross-sectional studies provided they met the eligible criteria [2,8]. In addition, our study provides the first data on the long-term risk for glucose intolerance in women with GDM based on the IADPSG criteria. However, our study has several limitations. Due to the language barrier, we only searched studies published in English, French, and Spanish. However, we only had to exclude one study due to the language used (Chinese). Several key variables, such as BMI and data on the family history of GDM and family history of T2DM, were absent in many studies. We contacted the authors to limit the number of missing variables. The overall quality of the studies was low, often due to the lack of a matched control group. In addition, nearly one-third of the studies did not report the diagnostic criteria used for GDM. Moreover, since the IADPSG criteria have only been recommended since 2010, only five studies with GDM based on the IADPSG criteria were identified and none of the studies evaluating the risk for cardiovascular events used the IADPSG criteria. More studies with GDM based on the IADPSG criteria and with a longer follow-up are needed to increase the quality of evidence concerning the long-term metabolic risk.

## 5. Conclusions

Women with a history of GDM based on the IADPSG criteria have a similarly increased risk for postpartum glucose intolerance compared to GDM based on other diagnostic criteria and other screening strategies. Women with GDM have a seven-fold increased risk of developing T2DM, a two-fold increased risk of developing IGT, and about a 1.5 times increased risk for cardiovascular events later in life compared to NGT women during pregnancy. As the relative increase in risk for T2DM is highest within the first six years after delivery, screening and prevention strategies should be implemented within the first years after the index pregnancy.

## Figures and Tables

**Table 1:** The characteristics of included studies: (**A**) study and patient characteristics, (**B**) characteristics concerning GDM, T2DM, and prediabetes, and (**C**) characteristics concerning stroke and myocardial infarction.

**A.**								
	**Study Type, Country**	**Ethnic Origin**	**Age at Pregnancy, yr** **Age at Follow-Up, yr** **(± SD or IQR)**	**BMI at Pregnancy, kg/m^2^** **BMI at Follow-Up, kg/m^2^** **(± SD or IQR)**		**Definition NGT Group**		**Duration of Follow-Up** **(± SD or IQR)**
Chodick et al. 2010 [37]	Retrospective cohort study, Israel	NR	NR 32.7 ± 5.5	NR 63.2% < 25		No matched controls		5.7 (± 4.0)
Pirkola et al. 2010 [38]	Prospective cohort study, Finland	NR	NR NR	NR NR		No matched controls		20.0
Akinci et al. 2011 [21]	Case-control study, Turkey	Caucasian	NR 31.9 ± 5.3	NR 27.1 ± 5.4		Controls (hospital staff) matched for age, and time period of pregnancy		3.4 (± 1.9)
Anderberg et al. 2011 [22]	Prospective cohort study, Sweden	Mixed	33.1 ± 4.9 NR	NR NR		Controls matched by random sampling for residency		1.3 (1.1–1.6)
Freibert et al. 2011 [54]	Cross-sectional study, USA (Kentucky)	NR	NR 57.1 ± 5.5	NR NR		No matched controls, women ≥ 50 years		NR
O’Reilly et al. 2011 [23]	Prospective cohort study, Ireland	Caucasian	NR 33.5 ± 4.7	NR NR		Controls matched for residency, and time period of pregnancy		0.23
Xiang et al. 2011 [39]	Retrospective cohort study, USA (California)	Mixed	NR 32.4 ± 5.2	NR NR		Five matched controls for each GDM by random sampling for ethnicity, age, and calendar year of study entry		3.9–5.2 (IQR NR)
Anderberg et al. 2012 [40]	Case-control study, Sweden	NR	NR NR	NR NR		Two matched controls for each GDM for year of birth, year of delivery, and municipality of residence		8.0–14.0
Sokup et al. 2012 [55]	Cross-sectional study, Poland	Caucasian	NR 29.0 (26.0–35.0)	NR 23.7 (21.0–27.5)		No matched controls		0.17–2
Tehrani et al. 2012 [41]	Case-control study, Iran	Middle-Eastern	NR 33.6 ± 7.5	NR 30.0 ± 4.7		Matched controls for age, and BMI from the TLGS cohort		9.0
Wang et al. 2012 [42]	Prospective cohort study, USA (Louisiana)	Mixed	NR 26.8 ± 0.2	NR 48.2 ± 1.7		Controls matched for age, and time period of pregnancy		8.6 (± NR)
Barden et al. 2013 [43]	Case-control study, Australia	Mixed	31.3 ± 0.4 (high risk), 33.8 ± 0.5 (low risk) NR	35.5 ± 5.0 (high risk), 27.3 ± 4.1 (low risk) NR		No matched controls		10.0
Hummel et al. 2013 [24]	Prospective cohort study, Germany	NR	NR	NR NR		No matched controls		5.5 (IQR NR)
Moleda et al. 2013 [25]	Prospective cohort study, Poland	Caucasian	30.8 ± 5.7 NR	22.4 ± 3.4 25.5 ± 5.6		Controls matched for age, and time period of delivery		7.4 (± 0.7)
Huopio et al. 2014 [17]	Prospective cohort study, Finland	Caucasian	32.0 ± 5.9 NR	26.4 ± 4.8 28.4 ± 5.5		No matched controls		7.3 (± 5.1)
McKenzie-Sampson et al. 2018 [56]	Retrospective cohort study, Canada	NR	NR NR	NR NR		No matched controls		NR
Kramer et al. 2014 [57]	Prospective cohort study, Canada	Mixed	35 (33–38) NR	NR 25.4 (22.4–30.1)		No matched controls		2.98 (± NR)
Mai et al. 2014 [44]	Case-control study, China	NR	NR 33.1 ± 4.8	NR 22.7 ± 3.5		No matched controls		2.5 (± 1.8)
Ajala et al. 2015 [26]	Retrospective cohort study, Canada	Caucasian	NR 39.2 ± 4.1	NR 28.9 ± 6.6		No matched controls		7.1 (± 1.6)
Cormier et al. 2015 [27]	Prospective cohort study, Canada	Caucasian	NR 36.4 ± 4.9	NR 27.7 ± 6.5		No matched controls		3.5 (± 2.0)
Kaul et al. 2015 [45]	Retrospective cohort study, Canada	NR	NR NR	NR NR		No matched controls		5.3 (2.2–8.4)
Lekva et al. 2015 [28]	Prospective cohort study, Norway	Caucasian	33.4 ± 4.54 NR	28.1 (26.7–30.1) 22.6 (22.4–27.9)		No matched controls		5.0
Pintaudi et al. 2015 [46]	Retrospective cohort study, Italy	NR	NR NR	NR NR		Three matched controls for each GDM for propensity scores		5.4 (2.9–7.3)
Sreelakshmi et al. 2015 [47]	Retrospective cohort study, India	Asian	NR NR	NR 24.6 ± 3.9		No matched controls		4.0
Vigneault et al. 2015 [29]	Cross-sectional study, Canada	Caucasian	NR NR	NR NR		No matched controls		3.9 (± NR)
Cho et al. 2016 [48]	Retrospective cohort study, South Korea	NR	NR NR	NR NR		No matched controls		8.0
Domínguez-Vigo et al. 2016 [49]	Case-control study, Spain	NR	33.8 ± 4.9 NR	NR NR		No matched controls		12.9 (± 0.4)
Goueslard et al. 2016 [20]	Retrospective cohort study, France	NR	NR NR	NR NR		No matched controls		7.0
Noctor et al. 2016 [30]	Prospective cohort study, Ireland	Caucasian	34.0 ± 5.0 NR	31.3 ± 6.6 29.7 ± 6.9		No matched controls		2.6 (± NR)
Sina et al. 2016 [50]	Prospective cohort study, Australia	Pacific Islanders	27.0 ± 6.7 NR	NR NR		No matched controls		11.9 (7.3–17.0)
Bond et al. 2017 [51]	Retrospective cohort study, Canada	Mixed	NR NR	NR NR		Controls matched for age, birth year, and residency		12.5 (± 5.6)
Gadgil et al. 2017 [31]	Cross-sectional study, USA	Asian	NR 51.1 ± 7.0	NR 26.7 ± 3.8		No matched controls, no self-reported GDM		NR
Herath et al. 2017 [52]	Retrospective cohort study, Sri Lanka	Asian	31.7 ± 5.4 NR	NR NR		No matched controls, no self-reported GDM		10.9 (± 0.35)
Minooee et al. 2017 [32]	Prospective cohort study, Iran	NR	NR 36.5 ± 8.0	NR 28.4 ± 4.5		No matched controls		12.1 (8.1–13.5)
Retnakaran et al. 2017 [3]	Retrospective cohort study, Canada	NR	NR NR	NR NR		No matched controls		10.0 (IQR NR)
Simmons et al. 2017 [33]	Cross-sectional study, New Zealand	Pacific Islanders	NR NR	NR NR		No matched controls, no self-reported GDM		NR
Tobias et al. 2017 [19]	Prospective cohort study, USA	Caucasian	27.5 ± 4.8 NR	21.5 ± 3.6 25.8 ± 5.9		No matched controls, no self-reported GDM		25.7 (± NR)
Daly et al. 2018 [18]	Retrospective cohort study, UK	NR	NR NR	NR NR		Four matched controls for each GDM for age, and timing of pregnancy		2.9 (IQR NR)
Gunderson et al. 2018 [53]	Prospective cohort study, USA	NR	NR NR	NR NR		No matched controls, no self-reported GDM		24.7 (± 6.6)
Huvinen et al. 2018 [34]	Prospective cohort study, Finland	NR	NR NR	NR NR		No matched controls		5.0 (4.0–6.0)
Lowe et al. 2018 [11]	Prospective cohort study, International	Mixed	NR 43.6 ± 5.4	29.7 ± 5.2 28.9 ± 6.5		No matched controls		11.4 (10.6–2.2)
Shen et al. 2018 [35]	Prospective cohort study, China	NR	NR 30.1 ± 3.5	NR 24.2 ± 3.9		No matched controls		3.5 (± NR)
Sudasinghe et al. 2018 [36]	Prospective cohort study, Sri Lanka	Asian	NR NR	NR NR		No matched controls		1.0
**B.**								
	**Screening Strategy for GDM**	**Criteria for GDM**	**Criteria for T2DM**	**Postpartum Screening Method for T2DM**	**GDM T2DM/Total** **NGT T2DM/Total** **(AR, %)**	**Criteria for Prediabetes**	**Postpartum Screening Method for Prediabetes**	**GDM Prediabetes/Total** **NGT Prediabetes/Total** **(AR, %)**
Chodick et al. 2010 [37]	Universal two-step screening	CC	NR	NR	1769/11,270 (15.7) 1741/174,146 (1.0)	-	-	-
Pirkola et al. 2010 [38]	Selective screening based on risk factors	NR	NR	NR	15/104 (14.4) 29/981 (3.0)	-	-	-
Akinci et al. 2011 [21]	Universal two-step screening	CC	ADA	OGTT	27/195 (13.8) 0/71 (0.0)	ADA	OGTT	101/195 (51.8) 2/71 (2.8)
Anderberg et al. 2011 [22]	NR	Local (Swedish) *	WHO	OGTT		WHO	OGTT	
Freibert et al. 2011 [54]	NR	NR	-	-	-	-	-	-
O’Reilly et al. 2011 [23]	Universal one-step screening	IADPSG	ADA	OGTT	9/300 (3.0) 0/220 (0.0)	ADA	OGTT	48/300 (16.0) 6/220 (2.7)
Xiang et al. 2011 [39]	NR	CC	ADA	OGTT or HbA1c	1539/12,998 (11.8) 1118/64,668 (1.7)	-	-	-
Anderberg et al. 2012 [40]	NR	NR	NR	NR	180/579 (31.1) 13/1131 (1.1)	-	-	-
Sokup et al. 2012 [55]	Universal two-step screening	WHO, 1999	-	-	-	WHO	OGTT	28/85 (32.9) 0/40 (0.0)
Tehrani et al. 2012 [41]	Universal one-step screening	IADPSG	ADA	OGTT	8/29 (27.6) 5/58 (8.6)	ADA	OGTT	4/29 (13.8) 9/58 (15.5)
Wang et al. 2012 [42]	NR	WHO, 1999	WHO	OGTT or FPG	327/1142 (28.6) 1067/18,856 (5.7)	-	-	-
Barden et al. 2013 [43]	Universal one-step screening	ADIPS	NR	FPG	20/112 (17.9) 0/48 (0.0)	-	-	-
Hummel et al. 2013 [24]	Selective screening based on risk factors	GDA	GDA	OGTT, FPG or HbA1c	8/102 (7.8) 0/15 (0.0)	NR	OGTT	37/105 (35.2) 1/16 (6.3)
Moleda et al. 2013 [25]	Universal one-step screening	Local (Polish) **	WHO	OGTT	13/199 (6.5) 0/50 (0.0)	WHO	OGTT	73/199 (36.7) 6/50 (12.0)
Huopio et al. 2014 [17]	Universal one-step screening	Local (Finnish) ***	ADA	OGTT	28/489 (5.7) 1/385 (0.3)	ADA	OGTT	233/489 (47.6) 100/385 (26.0)
Kramer et al. 2014 [57]	Universal two-step screening	NDDG	CDA	OGTT	7/105 (6.7) 2/232 (0.9)	CDA	OGTT	31/105 (29.5) 29/232 (12.5)
Mai et al. 2014 [44]	NR	ADA	ADA	OGTT	19/190 (10.0) 0/80 (0.0)	-	-	-
Ajala et al. 2015 [26]	Universal two-step screening	CDA	NR	OGTT or FPG	8/90 (8.9) 1/59 (1.7)	NR	OGTT or FPG	41/90 (45.6) 10/59 (16.9)
Cormier et al. 2015 [27]	NR	NR	CDA	OGTT	40/214 (18.7) 1/82 (1.2)	CDA	OGTT	121/214 (56.5) 11/82 (13.4)
Kaul et al. 2015 [45]	Universal two-step screening	CDA	NR	NR	1881/8731 (13.5) 3195/231,352 (1.4)	-	-	-
Lekva et al. 2015 [28]	Universal one-step screening	IADPSG	ADA	OGTT	1/52 (1.9) 0/248 (0.0)	ADA	OGTT	8/52 (15.4) 10/248 (4.2)
Pintaudi et al. 2015 [46]	Selective screening based on risk factors	ADA	NR	NR	773/3851 (20.1) 128/11,553 (1.1)	-	-	-
Sreelakshmi et al. 2015 [47]	NR	NR	NR	NR	6/60 (10.0) 1/120 (0.8)	-	-	-
Vigneault et al. 2015 [29]	NR	NR	CDA	OGTT	40/216 (10.5) 1/83 (1.2)	CDA	OGTT	122/216 (56.5) 11/83 (13.3)
Cho et al. 2016 [48]	NR	NR	NR	NR	249/2962 (8.4) 1642/50,147 (3.3)	-	-	-
Domínguez-Vigo et al. 2016 [49]	Universal two-step screening	NDDG, 1979	ADA	NR	41/308 (13.3) 1/130 (0.8)	-	-	-
Goueslard et al. 2016 [20]	NR	NR	NR	NR	1266/62,958 (2.0) 1674/1,452,429 (0.1)	-	-	-
Noctor et al. 2016 [30]	Universal one-step screening	IADPSG	ADA	OGTT	6/270 (2.2) 0/388 (0.0)	ADA	OGTT	64/270 (23.7) 14/388 (3.6)
Sina et al. 2016 [50]	NR	NR	WHO	NR	21/53 (39.6) 18/179 (10.1)	-	-	-
Bond et al. 2017 [51]	NR	CDA	NR	NR	6147/34,686 (17.7) 472/34,686 (1.4)	-	-	-
Gadgil et al. 2017 [31]	NR	NR	NR	OGTT	14/40 (35.0) 73/374 (19.5)	NR	OGTT	11/40 (27.5) 115/374 (30.7)
Herath et al. 2017 [52]	NR	WHO, 1999	WHO	OGTT or FPG	73/119 (61.3) 14/240 (5.8)	-	-	-
Minooee et al. 2017 [32]	Universal one-step screening	WHO, 1999	ADA	OGTT	49/476 (10.3) 93/1982 (4.7)	ADA	OGTT	279/476 (58.6) 877/1982 (44.2)
Retnakaran et al. 2017 [3]	Universal two-step screening	CDA	NR	NR	15,585/56,884 (24.4) 49,397/1,458,195 (3.4)	-	-	-
Simmons et al. 2017 [33]	Universal one-step screening	ADIPS	WHO	OGTT	6/52 (11.5) 100/2582 (3.9)	WHO	OGTT	4/52 (7.9) 209/2582 (8.1)
Tobias et al. 2017 [19]	NR	NR	NR	NR	1008/5292 (19.0) 4078/84,187 (4.8)	-	-	-
Daly et al. 2018 [18]	NR	NR	NR	NR	895/9118 (9.8) 142/37,281 (0.4)	-	-	-
Gunderson et al. 2018 [53]	NR	NR	ADA	OGTT, FPG or HbA1c	56/155 (36.1) 126/1083 (11.6)	-	-	-
Huvinen et al. 2018 [34]	Universal one-step screening	CC	NR	OGTT	9/179 (5.0) 3/154 (1.9)	NR	OGTT	29/179 (16.2) 11/154 (7.1)
Lowe et al. 2018 [11]	Universal one-step screening	IADPSG	ADA	OGTT	71/663 (10.7) 63/3946 (1.6)	ADA	OGTT	200/508 (39.4) 728/3945 (18.5)
McKenzie-Sampson et al. 2018 [56]	Universal two-step screening	NR	-	-	-	-	-	-
Shen et al. 2018 [35]	Universal two-step screening	WHO, 1999	ADA	OGTT	114/1263 (9.0) 7/705 (1.0)	ADA	OGTT	401/1080 (34.0) 198/698 (28.4)
Sudasinghe et al. 2018 [36]	NR	WHO, 1999	WHO	OGTT	11/59 (18.6) 3/57 (5.3)	WHO	OGTT	17/59 (28.9) 3/57 (5.3)
**C.**								
		**GDM Stroke/Total** **NGT Stroke/Total (AR, %)**				**GDM MI/Total** **NGT MI/Total (AR, %)**		
Freibert et al. 2011 [54]		- -				5/146 (3.42) 46/2558 (1.80)		
Goueslard et al. 2016 [20]		71/62,958 (0.11) 1181/1,452,429 (0.08)				26/62 958 (0.04) 257/1,452,429 (0.02)		
Tobias et al. 2017 [19]		33/5292 (0.62) 520/84,187 (0.62)				49/5292 (0.93) 563/84,187 (0.67)		
Daly et al. 2017 [18]		14/9118 (0.15) 50/37,281 (0.13)				14/9118 (0.15) 22/37,281 (0.06)		
McKenzie-Sampson et al. 2018 [56]		181/67,356 (0.27) 2207/1,003,311 (0.22)				280/67 356 (0.42) 2219/1,003,311 (0.22)		

GDM: gestational diabetes mellitus; MI: myocardial infarction; NGT: normal glucose tolerance; T2DM: type 2 diabetes mellitus; No matched controls: normal glucose tolerant women not matched by other characteristics to the GDM group; ADA: American Diabetes Association; ADIPS: Australasian Diabetes in Pregnancy Society; CC: Carpenter and Coustan; CDA: Canadian Diabetes Association; GDA; German Diabetes Association; IADPSG: International Association of Diabetes and Pregnancy Study Groups; NDDG: National Diabetes Data Group; WHO: World Health Organization; NR: not reported; OGTT: Oral Glucose Tolerance Test; BG: blood glucose; PG: plasma glucose; FPG: fasting plasma glucose; AR: absolute risk; SD: standard deviation; IQR: interquartile range. * Local (Swedish): one abnormal value on a 2 h 75 g OGTT with the following values: 2 h BG ≥ 9.0 mmol/L (PG ≥ 10.0 mmol/L). ** Local (Polish): FPG ≥ 100 mg/dL and/or 2 h PG ≥ 140 mg/dL. *** Local (Finnish): one or more abnormal values on a 2 h 75 g OGTT with the following values: until September 2001—FPG > 4.8 mmol/L, 1 h BG > 10.0 mmol/L, and 2 h BG > 8.7 mmol/L; since September 2001—FPG > 4.8 mmol/L, 1 h PG > 11.2 mmol/L, and 2 h PG > 9.9 mmol/L.

**Table 2:** Subgroup analyses for T2DM and prediabetes: (**A**) subgroup analyses for T2DM and (**B**) subgroup analyses for prediabetes.

**A.**							
**Subgroup**	**Number of studies**	**Weight of subjects (%)**	**Relative risk (95% CI)**	***p* for RR**	**I^2^ (%)**	**τ** **^2^**	***p* for heterogeneity**
**Diagnostic criteria GDM**				**0.17** *			
IADPSG	5	7.6	6.45 (4.74, 8.77)	<0.00001	0	0.00	0.57
Other criteria	23	53.4	9.08 (6.96, 11.85)	<0.00001	98	0.21	<0.00001
NR	12	39.0	6.04 (3.61, 10.10)	<0.00001	99	0.68	<0.00001
**Screening strategy GDM**				**0.09** *			
Universal two-step	8	19.7	11.56 (7.50, 17.84)	<0.00001	99	0.21	0.0007
Universal one-step	11	18.0	4.66 (2.68, 8.10)	<0.00001	67	0.36	<0.00001
Selective	3	8.1	8.17 (2.45, 27.23)	0.0006	90	0.82	<0.0001
NR	18	54.2	7.44 (4.96, 11.15)	<0.00001	99	0.58	<0.00001
**Follow-up, yr**				**<0.00001** *			
<3	7	9.5	13.15 (5.60, 30.85)	<0.00001	51	0.54	0.05
3–<6	12	25.2	15.95 (14.53, 17.52)	<0.00001	38	0.00	0.09
6–<10	7	17.6	6.23 (2.54, 15.26)	<0.0001	99	1.07	<0.00001
10–<15	9	29.5	6.82 (5.33, 8.71)	<0.00001	96	0.09	<0.00001
≥20	3	11.8	3.75 (3.14, 4.48)	<0.00001	42	0.01	0.18
NR	2	6.4	2.10 (1.32, 3.32)	0.002	17	0.02	0.27
**Age during pregnancy, yr**				**<0.00001** *			
<30	2	7.8	3.93 (3.69, 4.19)	<0.00001	0	0.00	0.99
30–<35	9	9.1	11.50 (7.39, 17.90)	<0.00001	0	0.00	0.97
≥35	0	-	-	-	-	-	-
NR	29	83.1	7.43 (5.92, 9.32)	<0.00001	99	0.25	<0.00001
**Age postpartum, yr**				**<0.00001** *			
<30	1	4.4	5.06 (4.54, 5.64)	<0.00001	NA	NA	NA
30–<35	7	14.4	12.98 (7.89, 21.35)	<0.00001	60	0.19	0.02
≥35	5	13.4	3.69 (1.76, 7.77)	0.0006	88	0.49	<0.00001
NR	27	67.8	7.87 (6.07, 10.21)	<0.00001	99	0.27	<0.00001
**BMI in pregnancy, kg/m^2^**				**<0.00001** *			
<25	1	4.4	3.93 (3.69, 4.19)	<0.00001	NA	NA	NA
25–<30	3	5.3	6.97 (5.05, 9.63)	<0.00001	0	0.00	0.40
≥30	2	1.0	18.20 (2.46, 134.42)	0.004	0	0.00	0.98
NR	34	89.3	7.51 (6.03, 9.36)	<0.00001	99	0.25	<0.00001
**BMI follow-up, kg/m^2^**				**<0.00001** *			
<25	5	9.0	15.64 (14.68, 16.66)	<0.00001	0	0.00	0.69
25–<30	11	21.6	4.21 (2.84, 6.25)	<0.00001	76	0.18	<0.00001
≥30	2	6.6	5.03 (4.52, 5.61)	<0.00001	0	0.00	0.38
NR	22	62.8	8.63 (6.65, 11.22)	<0.00001	99	0.25	<0.00001
**Ethnicity**				**0.02** *			
Caucasian	10	10.5	7.59 (4.06, 14.20)	<0.00001	23	0.22	0.23
Asian	4	9.8	4.87 (1.44, 16.41)	0.01	89	1.22	<0.00001
Pacific Islander	2	6.2	3.59 (2.29, 5.62)	<0.00001	0	0.00	0.56
Middle Eastern	1	2.2	3.20 (1.15, 8.92)	0.03	NA	NA	NA
Mixed	7	18.5	10.76 (5.55, 20.85)	<0.00001	98	0.53	<0.00001
NR	16	52.8	8.09 (6.06, 10.80)	<0.00001	99	0.26	<0.00001
**Diagnostic criteria T2DM**				**0.28** *			
WHO	7	16.9	5.27 (3.62, 7.66)	<0.00001	54	0.10	0.04
ADA	13	24.9	7.94 (4.22, 14.94)	<0.00001	88	0.79	<0.00001
CDA	3	3.2	11.31 (4.01, 31.91)	<0.00001	0	0.00	0.78
GDA	1	0.5	2.64 (0.16, 43.56)	0.50	-	-	-
NR	16	54.5	8.40 (6.29, 11.22)	<0.00001	99	0.27	<0.00001
**Screening method T2DM**				**0.23** *			
OGTT	19	30.6	5.42 (3.43, 8.55)	<0.00001	71	0.47	<0.00001
FGP	1	0.5	17.78 (1.10, 288.10)	0.04	NA	NA	NA
HbA1c	0	-	-	-	-	-	-
Multiple methods	6	16.8	7.23 (3.78, 13.84)	<0.00001	93	0.45	<0.00001
NR	14	52.1	9.38 (6.99, 12.58)	<0.00001	99	0.27	<0.00001
**B.**							
**Subgroup**	**Number of studies**	**Weight of subjects (%)**	**Relative risk (95% CI)**	***p* for RR**	**I^2^ (%)**	**τ** **^2^**	***p* for heterogeneity**
**Diagnostic criteria GDM**				**0.73** *			
IADPSG	5	22.7	2.79 (1.30, 5.96)	0.008	84	0.52	<0.00001
Other criteria	12	59.5	2.03 (1.57, 2.62)	<0.00001	82	0.10	<0.00001
NR	4	17.7	2.24 (0.86, 5.80)	0.10	83	0.72	0.0004
**Screening strategy GDM**				**0.63** *			
Universal two-step	5	23.6	3.22 (1.46, 7.10)	0.004	89	0.57	<0.00001
Universal one-step	10	53.0	2.18 (1.60, 2.97)	<0.00001	89	0.15	<0.00001
Selective	1	1.4	5.64 (0.83, 38.28)	0.08	NA	NA	NA
NR	5	22.0	2.63 (1.24, 5.57)	0.01	79	0.52	0.0007
**Follow-up, yr**				**0.04** *			
<3	6	26.5	4.16 (2.46, 7.03)	<0.00001	66	0.25	0.01
3–<6	7	29.8	3.10 (1.40, 6.87)	0.005	90	0.84	<0.00001
6–<10	4	21.8	2.00 (1.55, 2.58)	<0.00001	15	0.01	0.32
10–<15	2	16.9	1.68 (1.05, 2.68)	0.03	97	0.11	<0.00001
≥20	0	-	-	-	-	-	-
NR	2	5.0	0.31 (0.02, 5.36)	0.42	86	3.66	0.008
**Age during pregnancy, yr**				**0.66** *			
<30	0	-	-	-	-	-	-
30–<35	6	32.1	2.66 (1.68, 4.21)	<0.0001	77	0.21	0.0005
≥35	0	-	-	-	-	-	-
NR	15	67.9	2.36 (1.76, 3.16)	<0.00001	89	0.18	<0.00001
**Age postpartum, yr**				**0.20** *			
<30	1	0.7	27.17 (1.70, 434.17)	0.02	NA	NA	NA
30–<35	4	18.4	3.26 (0.94, 11.37)	0.06	91	1.40	<0.00001
≥35	5	29.8	1.93 (1.26, 2.96)	0.002	93	0.16	<0.00001
NR	11	51.2	2.73 (1.91, 3.88)	<0.00001	71	0.20	0.0002
**BMI in pregnancy, kg/m^2^**				**0.0003** *			
<25	0	-	-	-	-	-	-
25–<30	3	17.3	1.98 (1.61, 2.42)	<0.00001	52	0.01	0.12
≥30	1	5.9	6.57 (3.76, 11.47)	<0.00001	NA	NA	NA
NR	17	76.8	2.46 (1.82, 3.33)	<0.00001	86	0.23	<0.00001
**BMI follow-up, kg/m^2^**				**0.14** *			
<25	2	9.1	0.95 (0.31, 2.96)	0.93	27	0.38	0.24
25–<30	11	58.1	2.61 (1.88, 3.63)	<0.00001	91	0.19	<0.00001
≥30	1	3.4	1.25 (0.45, 3.48)	0.67	NA	NA	NA
NR	7	29.4	3.06 (1.97, 4.77)	<0.00001	52	0.17	0.05
**Ethnicity**				**<0.00001** *			
Caucasian	10	44.1	4.04 (2.43, 6.72)	<0.00001	83	0.45	<0.00001
Asian	2	4.2	0.71 (0.01, 68.22)	0.88	94	10.20	<0.0001
Pacific Islander	1	3.7	0.95 (0.37, 2.46)	0.92	NA	NA	NA
Middle Eastern	1	3.4	1.25 (0.45, 3.48)	0.67	NA	NA	NA
Mixed	3	21.1	2.16 (1.92, 2.44)	<0.00001	0	0.00	0.80
NR	4	23.5	1.32 (1.12, 1.56)	0.001	53	0.01	0.09
**Diagnostic criteria prediabetes**				**0.33** *			
WHO	5	17.9	2.76 (1.42, 5.36)	0.003	58	0.30	0.05
ADA	9	50.1	2.19 (1.60, 2.98)	<0.00001	92	0.14	<0.00001
CDA	3	18.4	3.39 (2.22, 5.17)	<0.00001	49	0.07	0.14
GDA	0	-	-	-	-	-	-
NR	4	13.5	1.55 (0.48, 5.04)	0.46	81	1.02	0.001
**Screening method prediabetes**				**0.63** *			
OGTT	20	94.4	2.44 (1.90, 3.13)	<0.00001	88	0.18	<0.00001
FGP	0	-	-	-	-	-	-
HbA1c	0	-	-	-	-	-	-
Multiple methods	1	5.6	2.69 (1.46, 4.94)	0.001	NA	NA	NA
NR	0	-	-	-	-	-	-

BMI: body mass index (kg/m^2^); NR: not reported; RR: relative risk; 95% CI: confidence interval; NA: not applicable; WHO: World Health Organization; ADA: American Diabetes Association; CDA: Canadian Diabetes Association; GDA: German Diabetes Association. * *p* values for test for subgroup differences. I^2^ represents the total between-studies variability, τ^2^ represents the between-studies variance between studies, and *p* for heterogeneity represents the *p* values for the heterogeneity within subgroups.

## Supplementary Materials

Supplementary materials can be found at https://www.mdpi.com/2077-0383/8/9/1431/s1. Table S1: Detailed search strategy; Table S2: Sensitivity analyses; Table S3: Assessment of quality of studies by NOS; Figure S1: Funnel plot.
