# Supplementary material for: The Risk for Glucose Intolerance after Gestational Diabetes Mellitus since the Introduction of the IADPSG Criteria: A Systematic Review and Meta-Analysis
Source: J Clin Med. 2019 Sep 10;8(9):1431. doi: 10.3390/jcm8091431 (PMC6780861; doi:10.3390/jcm8091431)
Supplement: Supplementary file 1 [file jcm-08-01431-s001.pdf]

Journal of
Clinical Medicine

MDPI

Supplementary Material

Supplementary Table S1. Detailed search strategy.

1. Search string for GDM	"Diabetes, Gestational"[Mesh:NoExp] OR "Pregnancy-Induced Diabetes"[Tiab] OR "Pregnancy Induced Diabetes"[Tiab] OR "Gestational Diabetes"[Tiab] OR "Hyperglycemia In Pregnancy"[Tiab] OR "Hyperglycaemia In Pregnancy"[Tiab] OR "GDM"[Tiab] OR "Diabetic Pregnancy"[Tiab] OR "Diabetes Gravidarum"[Tiab] OR "Pregnancy Diabetes"[Tiab] OR "Dysglycemia In Pregnancy"[Tiab] OR "Dysglycaemia In Pregnancy"[Tiab]
2. Search string for T2DM	"Diabetes Mellitus, Type 2"[Mesh:NoExp] OR "Noninsulin-Dependent Diabetes"[Tiab] OR "Noninsulin Dependent Diabetes"[Tiab] OR "Non Insulin Dependent Diabetes"[Tiab] OR "Non-Insulin-Dependent Diabetes"[Tiab] OR "NIDDM"[Tiab] OR "Type II Diabetes"[Tiab] OR "Type 2 Diabetes"[Tiab] OR "Ketosis-Resistant Diabetes"[Tiab] OR "Ketosis Resistant Diabetes"[Tiab] OR "Slow-Onset Diabetes"[Tiab] OR "Slow Onset Diabetes"[Tiab] OR "Adult-Onset Diabetes"[Tiab] OR "Adult Onset Diabetes"[Tiab] OR "Insulin Independent Diabetes"[Tiab] OR "Insulin-Independent Diabetes"[Tiab]
3. Search string for prediabetes	"Prediabetic State"[Mesh] OR Prediabetic State*[Tiab] OR Pre-Diabetic State*[Tiab] OR Prediabetic Stage*[Tiab] OR Pre-Diabetic Stage*[Tiab] OR "Prediabetes"[Tiab] OR "Pre-Diabetes"[Tiab] OR "Impaired Fasting Glycemia"[Tiab] OR "Impaired Fasting Glycaemia"[Tiab] OR "Intermediate Hyperglycemia"[Tiab] OR "Intermediate Hyperglycaemia"[Tiab] OR "Glucose Intolerance"[Mesh] OR Glucose Intolerance*[Tiab] OR "Impaired Glucose Tolerance"[Tiab] OR "Glucose Tolerance Impairment"[Tiab] OR "Abnormal Glucose Tolerance"[Tiab] OR "Glucose Tolerance Disorder"[Tiab]
4. Search string for stroke	"Stroke"[Mesh] OR Stroke*[Tiab] OR Cerebrovascular Accident*[Tiab] OR Cerebro Vascular Accident*[Tiab] OR Cerebro-Vascular Accident*[Tiab] OR Cerebral Vascular Accident*[Tiab] OR Cerebral Accident*[Tiab] OR CVA*[Tiab] OR Brain Accident*[Tiab] OR "Apoplexy"[Tiab] OR "Apoplexia"[Tiab] OR "Brain Ischemia"[Mesh:NoExp] OR "Brain Ischemia"[Tiab] OR "Brain Ischaemia"[Tiab] OR "Cerebral Ischemia"[Tiab] OR "Cerebral Ischaemia"[Tiab] OR Brain Infarct*[Tiab] OR Cerebral Infarct*[Tiab] OR Cerebral Vascular Infarct*[Tiab] OR Cerebrovascular Infarct*[Tiab] OR Cerebro Vascular Infarct*[Tiab] OR Cerebro-Vascular Infarct*[Tiab] OR Cortical Infarct*[Tiab] OR "Cerebral Artery Syndrome"[Tiab] OR "Cerebral Artery Embolus"[Tiab] OR "Cerebral Artery Occlusion"[Tiab] OR "Cerebral Artery Thrombosis"[Tiab] OR Cerebral Artery Infarct*[Tiab] OR ACA Infarct*[Tiab] OR MCA Infarct*[Tiab] OR PCA Infarct*[Tiab] OR Brain Stem Infarct*[Tiab] OR Brainstem Infarct*[Tiab] OR "Brain Stem Ischemia"[Tiab] OR "Brain Stem Ischaemia"[Tiab] OR "Brainstem Ischemia"[Tiab] OR "Brainstem Ischaemia"[Tiab] OR "Lacunar Syndrome"[Tiab] OR Lacunar Infarct*[Tiab] OR "Lacunar Ischemia"[Tiab] OR "Lacunar Ischaemia"[Tiab] OR "Ischemic Attack, Transient"[Mesh] OR Transient Ischemic Attack*[Tiab] OR Transient Ischaemic Attack*[Tiab] OR "TIA"[Tiab]
5. Search string for myocardial infarction	“Myocardial Ischemia”[Mesh:NoExp] OR “Myocardial Ischemia”[Tiab] OR “Myocardial Ischaemia”[Tiab] OR “Heart Muscle Ischemia”[Tiab] OR “Heart Muscle Ischaemia”[Tiab] OR Ischemic Heart Disease*[Tiab] OR Ischaemic Heart Disease*[Tiab] OR “Cardiac Ischemia”[Tiab] OR “Cardiac Ischaemia”[Tiab] OR “Cardiac Muscle Ischemia”[Tiab] OR “Cardiac Muscle Ischaemia”[Tiab] OR “Heart Ischemia”[Tiab] OR “Heart Ischaemia”[Tiab] OR “Acute Coronary Syndrome”[Mesh] OR Acute Coronary Syndrome*[Tiab] OR “Angina Pectoris”[Mesh:NoExp] OR Angina*[Tiab] OR “Angor”[Tiab] OR Stenocardia*[Tiab] OR “Angina, Stable”[Mesh] OR “Angina, Unstable”[Mesh:NoExp] OR “Coronary Disease”[Mesh:NoExp] OR Coronary Disease*[Tiab] OR Coronary Heart Disease*[Tiab] OR “Coronary Artery Disease”[Mesh] OR Coronary Artery Disease*[Tiab] OR “Coronary Arteriosclerosis”[Tiab] OR “Coronary Arterioscleroses”[Tiab] OR “Coronary Atherosclerosis”[Tiab] OR “Coronary Atheroscleroses”[Tiab] OR “Coronary Occlusion”[Mesh] OR Coronary Occlusion*[Tiab] OR Coronary Artery Occlusion*[Tiab] OR “Coronary Stenosis”[Mesh:NoExp] OR “Coronary Stenosis”[Tiab] OR “Coronary Stenoses”[Tiab] OR “Coronary Artery Stenosis”[Tiab] OR “Coronary Artery Stenoses”[Tiab] OR “Coronary Obstruction”[Tiab] OR “Coronary Artery Obstruction”[Tiab] OR “Coronary Thrombosis”[Mesh] OR “Coronary Thrombosis”[Tiab] OR “Coronary Thromboses”[Tiab] OR “Myocardial Infarction”[Mesh:NoExp] OR Heart Attack*[Tiab] OR Myocardial Infarct*[Tiab] OR Heart Infarct*[Tiab] OR Cardiac Infarct*[Tiab] OR Cardial Infarct*[Tiab] OR Heart Muscle Infarct*[Tiab] OR Cardiac Muscle Infarct*[Tiab] OR “Anterior Wall Myocardial Infarction”[Mesh] OR “Inferior Wall Myocardial Infarction”[Mesh] OR “Non-ST Elevated Myocardial Infarction”[Mesh] OR “NSTEMI”[Tiab] OR “ST Elevation Myocardial Infarction”[Mesh] OR “STEMI”[Tiab]

6. Search string for risk	"Prevalence"[Mesh] OR "Prevalence"[Tiab] OR "Incidence"[Mesh] OR "Incidence"[Tiab] OR "Risk"[Mesh:NoExp] OR "Risk Factors"[Mesh] OR "Risk Assessment"[Mesh] OR "Risk"[Tiab] OR "Odds Ratio"[Mesh] OR "Odds"[Tiab] OR "Prognosis"[Mesh:NoExp] OR Prognos*[Tiab] OR "Follow-Up Studies"[Mesh] OR "Follow-up"[Tiab] OR "Followup"[Tiab] OR "Follow Up"[Tiab] OR Predict*[Tiab] OR "Disease Progression"[Mesh:NoExp] OR "Disease Progression"[Tiab] OR "Disease Exacerbation"[Tiab]
7.	#2 OR #3 OR #4 OR #5
8.	#1 AND #7 AND #6
9.	Time restriction: Publication date from 2010/01/01.

GDM: gestational diabetes mellitus; T2DM: type 2 diabetes mellitus.

Table S2. Sensitivity analyses. A. T2DM; B. Prediabetes; C1. Stroke; C2. Myocardial infarction.

A.

Overall effect

	GDM	NGT		RR (95% CI)
Total (95% CI)	216,725	3,633,350	100.0%	7.42 [5.99, 9.19]
Total events	32,356	65,209		

Heterogeneity: $Tau^2 = 0.27$; $Chi^2 = 2843.10$, df = 39 (P < 0.00001); $I^2 = 99\%$
Test for overall effect: Z = 18.32 (P < 0.00001)

Sensitivity analysis for quality of the NOS: studies with good quality (9 out of 40 studies)

	GDM	NGT		RR (95% CI)
Total (95% CI)	50,230	104,015	100.0%	15.21 [8.36, 27.69]
Total events	8388	1822		

Heterogeneity: $Tau^2 = 0.53$; $Chi^2 = 377.36$, df =8 (P < 0.00001); $I^2 = 98\%$
Test for overall effect: Z = 8.91 (P < 0.00001)

Sensitivity analysis for the type of study: prospective cohort studies (19 out of 40 studies)

	GDM	NGT		RR (95% CI)
Total (95% CI)	11,277	113,917	100.0%	4.41 [3.64, 5.35]
Total events	1810	5491		

Heterogeneity: $Tau^2 = 0.05$; $Chi^2 = 57.92$, df = 18 (P < 0.00001); $I^2 = 69\%$
Test for overall effect: Z = 15.11 (P < 0.00001)

B.

Overall effect

	GDM	NGT		RR (95% CI)
Total (95% CI)	5003	11,891	100.0%	2.45 [1.92, 3.13]
Total events	1873	2365		

Heterogeneity: $Tau^2 = 0.18$; $Chi^2 = 167.96$, df = 20 (P < 0.00001); $I^2 = 88\%$
Test for overall effect: Z = 7.23 (P < 0.00001)

Sensitivity analysis for quality of the NOS: studies with good quality (4 out of 21 studies)

	GDM	NGT		RR (95% CI)
Total (95% CI)	854	508	100.0%	4.57 [2.13, 9.80]
Total events	260	30		

Heterogeneity: $Tau^2 = 0.41$; $Chi^2 = 10.41$, df =3 (P = 0.02); $I^2 = 71\%$
Test for overall effect: Z = 3.91 (P < 0.0001)

Sensitivity analysis for the type of study: prospective cohort studies (14 out of 21 studies)

	GDM	NGT		RR (95% CI)
Total (95% CI)	4296	8624	100.0%	2.41 [1.87, 3.10]
Total events	1571	2010		

Heterogeneity: $Tau^2 = 0.14$; $Chi^2 = 122.42$, df = 13 (P < 0.00001); $I^2 = 89\%$
Test for overall effect: Z = 6.85 (P < 0.00001)

C1.

Overall effect

	GDM	NGT		RR (95% CI)
Total (95% CI)	144,724	2,577,208	100.0%	1.23 [1.09, 1.38]
Total events	299	3958		

Heterogeneity: $Tau^2 = 0.00$; $Chi^2 = 2.25$, df =3 (P = 0.52); I^2 =0%

Test for overall effect: Z = 3.44 (P = 0.0006)

Sensitivity analysis for quality of the NOS: studies with good quality (1 out of 4 studies)

	GDM	NGT		RR (95% CI)
Total (95% CI)	9118	37,281	100.0%	1.14 [0.63, 2.07]
Total events	14	50		

Heterogeneity: Not applicable

Test for overall effect: Z = 0.45 (P = 0.65)

Sensitivity analysis for the type of study: prospective cohort studies (1 out of 4 studies)

	GDM	NGT		RR (95% CI)
Total (95% CI)	5292	84,187	100.0%	1.01 [0.71, 1.43]
Total events	33	520		

Heterogeneity: Not applicable

Test for overall effect: Z = 0.05 (P = 0.96)

C2.

Overall effect

	GDM	NGT		RR (95% CI)
Total (95% CI)	144,870	2,579,766	100.0%	1.85 [1.53, 2.24]
Total events	374	3107		

Heterogeneity: $Tau^2 = 0.02$; $Chi^2 = 6.17$, df =4 (P = 0.19); I^2 = 35%

Test for overall effect: Z = 6.30 (P < 0.00001)

Sensitivity analysis for quality of the NOS: studies with good quality (1 out of 5 studies)

	GDM	NGT		RR (95% CI)
Total (95% CI)	9118	37,281	100.0%	2.60 [1.33, 5.08]
Total events	14	22		

Heterogeneity: Not applicable

Test for overall effect: Z = 2.80 (P = 0.005)

Sensitivity analysis for the type of study: prospective cohort studies (1 out of 5 studies)

	GDM	NGT		RR (95% CI)
Total (95% CI)	5292	84,187	100.0%	1.38 [1.04, 1.85]
Total events	49	563		

Heterogeneity: Not applicable

Test for overall effect: Z = 2.19 (P = 0.03)

GDM: gestational diabetes mellitus; NGT: normal glucose tolerance; NOS: the Newcastle-Ottawa Scale; RR: relative risk; 95% CI: 95% confidence interval.

Table S3. Assessment of quality of studies by NOS.

	Study design	Selection	Comparability	Outcome/Exposure	Overall score	Overall quality
Chodick et al. 2010 (37)	Cohort	★★★★	☆☆	★★☆	6	Poor
Pirkola et al. 2010 (38)	Cohort	★★★☆	☆☆	★★☆	5	Poor
Akinci et al. 2011 (21)	Case-control	★★★☆	★☆	★★☆	6	Good
Anderberg et al. 2011 (22)	Cohort	★★★☆	★☆	★★★	7	Good
Freibert et al. 2011 (54)	Cross-sectional	★★☆☆	★☆	★★☆	4	Fair
O'Reilly et al. 2011 (23)	Cohort	★★★☆	★☆	★★★	7	Good
Xiang et al. 2011 (39)	Cohort	★★★★	★☆	★★★	8	Good
Anderberg et al. 2012 (40)	Case-control	★★☆☆	★☆	★☆☆	4	Poor
Sokup et al . 2012 (55)	Cross-sectional	★★☆☆☆	★☆	★★★	6	Fair
Tehrani et al. 2012 (41)	Case-control	★★☆☆	★☆	★★☆	5	Fair
Wang et al. 2012 (42)	Cohort	★★★★	★☆	★★★	8	Good
Barden et al. 2013 (43)	Case-control	★☆☆☆	☆☆	★☆☆	2	Poor
Hummel et al. 2013 (24)	Cohort	★★☆☆	☆☆	★★☆	4	Poor
Moleda et al. 2013 (25)	Cohort	★★★☆	★☆	★★☆	6	Good
Huopio et al. 2014 (17)	Cohort	★★★★	☆☆	★★☆	6	Poor
Kramer et al. 2014 (57)	Cohort	★★★☆	☆☆	★★★	6	Poor
Mai et al. 2014 (44)	Case-control	★★☆☆	☆☆	★★☆	4	Poor
Ajala et al. 2015 (26)	Cohort	★★★☆	☆☆	★★☆	5	Poor
Cormier et al. 2015 (27)	Cohort	★★★☆	☆☆	★★☆	5	Poor
Kaul et al. 2015 (45)	Cohort	★★★★	☆☆	★★★	7	Poor
Lekva et al. 2015 (28)	Cohort	★★★☆	☆☆	★★★	6	Poor
Pintaudi et al. 2015 (46)	Cohort	★★★★	★☆	★★★	8	Good
Sreelakshmi et al. 2015 (47)	Cohort	★★☆☆	☆☆	★☆☆	3	Poor
Vigneault et al. 2015 (29)	Cross-sectional	★☆☆☆☆	☆☆	★★☆	3	Poor
Cho et al. 2016 (48)	Cohort	★★★★	☆☆	★★★	7	Poor
Domínguez-Vigo et al. 2016 (49)	Case-control	★☆☆☆	☆☆	★★☆	3	Poor
Goueslard et al. 2016 (20)	Cohort	★★★★	☆☆	★★☆	6	Poor
Noctor et al. 2016 (30)	Cohort	★★★☆	☆☆	★★★	6	Poor
Sina et al. 2016 (50)	Cohort	★★☆☆	☆☆	★☆☆	3	Poor
Bond et al. 2017 (51)	Cohort	★★★★	★☆	★★☆	7	Good
Gadgil et al. 2017 (31)	Cross-sectional	★☆☆☆☆	☆☆	★★★	4	Poor
Herath et al. 2017 (52)	Cohort	★★☆☆	☆☆	★★☆	4	Poor
Minooee et al. 2017 (32)	Cohort	★★★★	☆☆	★★★	7	Poor
Retnakaran et al. 2017 (3)	Cohort	★★★★	☆☆	★★★	7	Poor
Simmons et al. 2017 (33)	Cross-sectional	☆☆☆☆☆	☆☆	★★☆	2	Poor
Tobias et al. 2017 (19)	Cohort	★★★★	☆☆	★★☆	6	Poor
Daly et al. 2018 (18)	Cohort	★★★★	★☆	★★★	8	Good
Gunderson et al. 2018 (53)	Cohort	★★★★	☆☆	★★★	7	Poor
Huvinen et al. 2018 (34)	Cohort	★★★☆	☆☆	★★☆	5	Poor
Lowe et al. 2018 (11)	Cohort	★★★★	☆☆	★★★	7	Poor
McKenzie-Samspon et al. 2018 (56)	Cohort	★★★★	☆☆	★★☆	6	Poor
Shen et al. 2018 (35)	Cohort	★★★☆	☆☆	★★★	6	Poor
Sudasinghe et al. 2018 (36)	Cohort	★★☆☆	☆☆	★★☆	4	Poor

NOS: the Newcastle-Ottawa Scale. A solid star represents an awarded star for that given domain. A white star represents a star that is not awarded for that given domain. Good quality: 3 or 4 stars for selection and 1 or 2 stars for comparability and 2 or 3 stars for outcome/exposure. Fair quality: 2 stars for selection and 1 or 2 stars for comparability and 2 or 3 stars for outcome/exposure. Poor quality: 0 or 1 star for selection or 0 stars for comparability or 0 or 1 stars in outcome/exposure. The term outcome in the third domain applies to cohort studies and cross-sectional studies. The term exposure in the third domain applies to case-control studies.

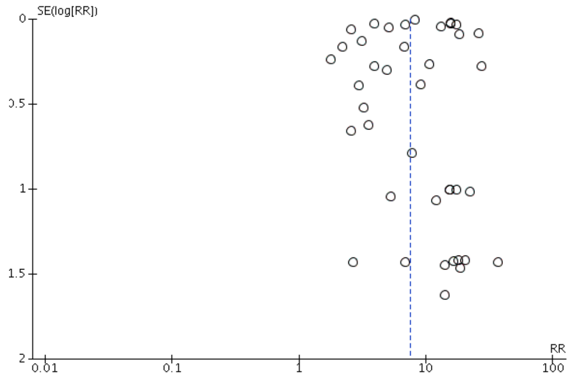

(**A**)

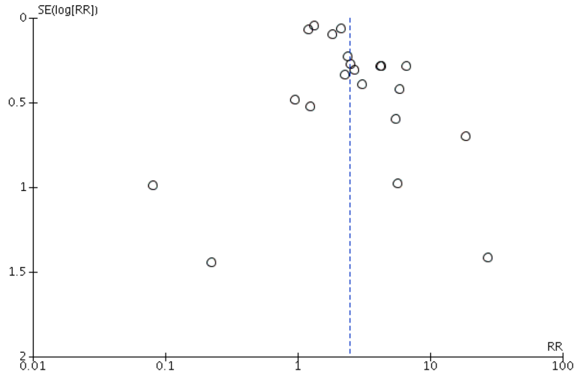

(**B**)

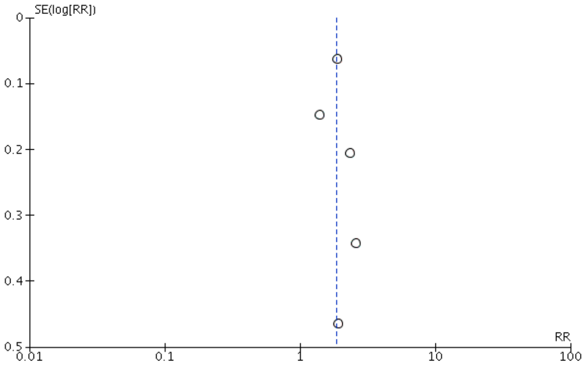

(**C**)

Figure S1. Funnel plot. (**A**) T2DM**;** (**B**) Prediabetes**;** (**C**) Stroke and Myocardial infarction. X-axis is log scale of relative risk (RR). Y-axis is standard error of log RR. Dotted horizontal line represents the pooled unadjusted RR.

© 2019 by the authors. Submitted for possible open access publication under the terms and conditions of the Creative Commons Attribution (CC BY) license (http://creativecommons.org/licenses/by/4.0/).
